# The Anti-Inflammatory and Immunomodulatory Activities of Natural Products to Control Autoimmune Inflammation

**DOI:** 10.3390/ijms24010095

**Published:** 2022-12-21

**Authors:** Kamal D. Moudgil, Shivaprasad H. Venkatesha

**Affiliations:** 1 Department of Microbiology and Immunology, University of Maryland School of Medicine, Baltimore, MD 21201, USA; 2 Baltimore VA Medical Center, Baltimore, MD 21201, USA; 3 Vita Therapeutics, Baltimore, MD 21201, USA

**Keywords:** autoimmunity, boswellic acids, colitis, curcumin, diabetes, EGCG, multiple sclerosis, natural products, psoriasis, resveratrol, rheumatoid arthritis, triptolide

## Abstract

Inflammation is an integral part of autoimmune diseases, which are caused by dysregulation of the immune system. This dysregulation involves an imbalance between pro-inflammatory versus anti-inflammatory mediators. These mediators include various cytokines and chemokines; defined subsets of T helper/T regulatory cells, M1/M2 macrophages, activating/tolerogenic dendritic cells, and antibody-producing/regulatory B cells. Despite the availability of many anti-inflammatory/immunomodulatory drugs, the severe adverse reactions associated with their long-term use and often their high costs are impediments in effectively controlling the disease process. Accordingly, suitable alternatives are being sought for these conventional drugs. Natural products offer promising adjuncts/alternatives in this regard. The availability of specific compounds isolated from dietary/medicinal plant extracts have permitted rigorous studies on their disease-modulating activities and the mechanisms involved therein. Here, we describe the basic characteristics, mechanisms of action, and preventive/therapeutic applications of 5 well-characterized natural product compounds (Resveratrol, Curcumin, Boswellic acids, Epigallocatechin-3-gallate, and Triptolide). These compounds have been tested extensively in animal models of autoimmunity as well as in limited clinical trials in patients having the corresponding diseases. We have focused our description on predominantly T cell-mediated diseases, such as rheumatoid arthritis, multiple sclerosis, Type 1 diabetes, ulcerative colitis, and psoriasis.

## 1. Introduction

Chronic inflammation is an integral component of autoimmune diseases, which are caused by dysregulation of the immune system, such that the host mounts an immune response to body’s own component proteins and/or cells [1,2,3,4,5]. A variety of anti-inflammatory and immunomodulatory drugs are available for these disorders [6,7,8,9,10]. However, prolonged use of these drugs, which is necessary to keep persistent autoimmune reactivity in check, is frequently associated with severe adverse reactions and unavoidable high expense for the drugs. Newer drugs, such as biologics, may cause unintended systemic immune suppression, which increases the risk of severe infections and possibly, certain malignancies [6,7,10]. Furthermore, a proportion of autoimmune patients, for example, about 30–40% in case of rheumatoid arthritis (RA), fail to respond well to these drugs [11]. For these reasons, increasing proportion of patients with autoimmune diseases are resorting to the use of natural products [12,13,14]. Most popular among these products are plant-derived natural products, which have been part of various traditional systems of medicine across the world. Traditional Chinese medicine (TCM), Ayurvedic medicine, and indigenous African medicine are examples of such systems [11,13,14,15,16,17,18,19].

Accordingly, a variety of plant extracts and their bioactive components have extensively been studied for their anti-inflammatory and immunomodulatory activities, and for their applications in the treatment of autoimmune diseases [12,13,14]. These natural products are first tested in various animal models of autoimmunity and subsequently in limited clinical trials in patients with those diseases. In this article, we have focused our discussion to 5 purified compounds derived from plant extracts. These include, Resveratrol [19,20,21,22,23,24,25,26], Curcumin [12,27,28,29,30,31], Boswellic acids [15,16,32,33], Epigallocatechin-3-gallate (EGCG) [34,35,36,37,38,39,40], and Triptolide [18,41,42,43,44,45,46].

We have described below the effects of these natural products on inflammation in predominantly T cell-driven autoimmune diseases: rheumatoid arthritis (RA), multiple sclerosis (MS), type 1 diabetes (T1D), ulcerative colitis (UC; colitis), and psoriasis. For space constraints, limited number of studies for each natural product compound are discussed below.

## 2. Autoimmune Diseases Result from a Breakdown of Self-Tolerance Leading to the Generation of Immune Response to Self-Antigens

Autoimmune diseases are manifestations of a series of events that involve an interplay between genetic and environmental factors, leading to a breakdown of self-tolerance and activation of potentially self-reactive T cells [4,5,47,48,49]. In healthy individuals, such T cells exist in the mature T cell repertoire, but their activation is kept in check by a variety of peripheral tolerance mechanisms [50]. These mechanisms include, T cell ignorance, lack of co-stimulation, activity of different types of regulatory T cells, and others [51,52]. A failure of these mechanisms results in uncontrolled activation of T cells directed against various self-antigens implicated in different autoimmune diseases [53]. These activated T cells then stimulate macrophages and/or provide help to the B cells, leading to propagation of anti-self immune responses. A variety of cytokines, chemokines and biochemical mediators of inflammation released by these immune cells trigger cascades of inflammatory pathways, tissue damage and dysfunction, and clinical manifestations [53,54]. Autoimmune diseases can either be organ-specific or systemic. Rheumatoid arthritis (RA), multiple sclerosis (MS), type 1 diabetes (T1D), ulcerative colitis (colitis) (a component of inflammatory bowel disease or IBD), and psoriasis are examples of major human autoimmune diseases [55,56,57,58,59,60] (Table 1). The organs most affected in these diseases include the synovial joints in RA, the central nervous system (CNS) in MS, the pancreatic β-islets in T1D, colon in UC, and skin in psoriasis [57,58,60,61,62]. In addition, there may be varying levels of effects on other organs, as commonly observed in RA.

## 3. The Pathogenesis of Autoimmunity Involves an Interplay between Genetic and Environmental Factors

The precise etiology of autoimmune diseases is not yet fully known. It is clear that there is genetic susceptibility to several autoimmune diseases [49,65]. The major histocompatibility complex (MHC) locus is one of the important genetic factors in this regard (Table 1). There also is evidence for environmental factors, among which microbial infections are considered to be of high significance [5,65]. Microbes can contribute to the activation of self-reactive T cells via different mechanisms, including upregulation of co-stimulation in antigen-presenting cells (APCs), enhanced processing of certain antigenic determinants under inflammatory conditions, and antigenic mimicry (e.g., conformational/sequence resemblance) between microbial and host antigenic determinants (molecular mimicry) [55,56,59,63,69]. The pathogenic T cells in autoimmune diseases target subsets of self-antigens. Multiple self-antigens have been implicated in each of the above-mentioned autoimmune diseases (RA, MS, T1D, colitis and psoriasis) [57,58,60,61,62,66,67,68,70] (Table 1).

## 4. Autoimmunity Is Characterized by an Imbalance of Cytokines and T Cell Subsets, and Aberrant Antibody Responses

**Figure 1:**
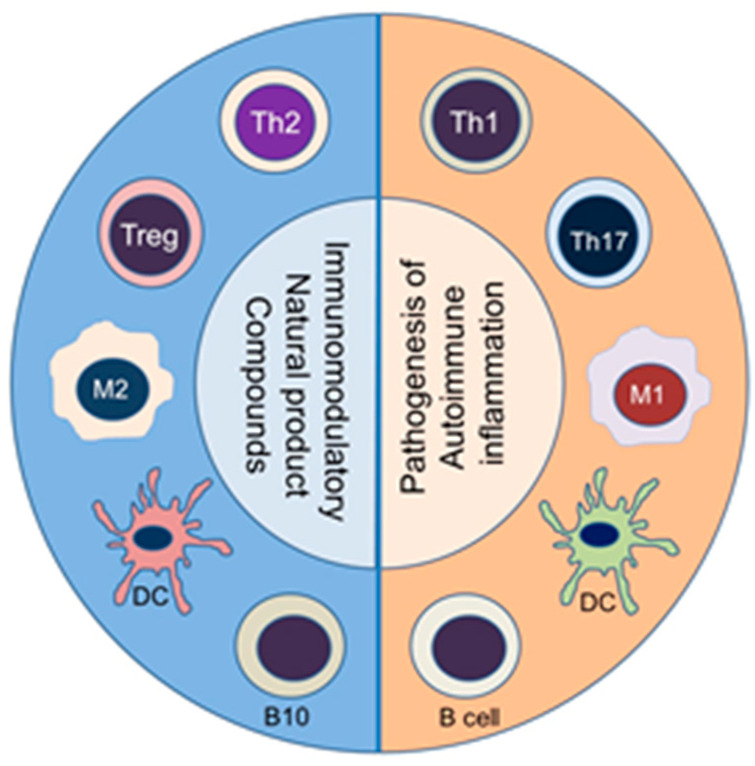
Immunomodulation by natural product compounds involves an altered balance between subsets of diverse immune cell types. The corresponding pathogenic (pro-inflammatory) (Right panel) versus protective (anti-inflammatory/immunomodulatory) (Left panel) immune cell subsets are shown: Th1 vs. Th2, Th17 vs. Treg, M1 vs. M2, activating vs. tolerogenic DC, and antibody-producing vs. regulatory B cells, respectively. (Abbreviations (Top to bottom)): Th = T helper cell; Treg = T regulatory cells; M1 = M1-type macrophage; M2 = M2-type macrophage; DC = dendritic cells (Right panel, activating DC; Left panel, tolerizing DC); B10 = B cells secreting IL-10 (a regulatory B cell subset); B cell = activated B cell for antibody production.

Naïve T cells that are activated by a particular self-antigen can acquire different phenotypes depending on the local milieu, particularly the type of cytokines present. Accordingly, naïve T cells can differentiate into distinct T helper (Th) type, such as Th1 or Th17 cells, which can mediate pathogenic effector functions [53,71,72]. Under another set of conditions, a naïve T cell can develop into Th2 or T regulatory cells (Treg), which can control the activity of pathogenic Th cells [53,64]. The Th cells secrete prototypic cytokines and express a distinct transcription factor [72]. Among the cytokines, interferon-γ (IFN-γ) and IL-17 are shown to promote inflammation, whereas IL-4 and IL-10 inhibit inflammation [52]. An imbalance between pro-inflammatory and anti-inflammatory cytokines, as well as that between pathogenic (Th1/Th17) and protective (Th2/Treg, respectively) T cells have been reported in various autoimmune diseases [64,73,74]. A similar imbalance can occur in macrophage subsets, such as pro-inflammatory M1 macrophages versus anti-inflammatory M2 macrophages; in dendritic cells (DCs), such as activating DCs versus tolerogenic DCs; and in B cells, such as antibody-producing B cells versus regulatory B cells that secrete IL-10 (known as B10 cells) (Figure 1). Accordingly, a deliberate re-setting of the balance between these cytokines (e.g., IFN-γ/IL-4 balance) and immune cell subsets (e.g., Th17/Treg balance) is one of the approaches under development for the treatment of these diseases [75]. This principle is also being exploited by using natural products compounds (Figure 1).

A dysregulation of cytokines and Th cells in turn can influence B cell activation and the type of antibody isotype produced. Thus, both cellular and humoral arms of the immune response can be affected in this manner. For simplicity, autoimmune diseases may be classified as T cell-mediated or antibody-mediated, but in reality, the disease manifestation involves the contribution of both arms of the immune system to different extents. For example, T1D is characterized by anti-insulin antibodies. In RA, rheumatoid factors (RFs) have long served as a disease marker, but their pathogenic significance is not fully validated. However, there is a correlation between smoking and RA, which involves anti-citrullinated protein antibodies (ACPAs). Here, smoking has been invoked in inducing citrullination, a type of post-translational modification, of certain self-antigens in which an arginine residue is converted into citrulline [76]. This renders that protein immunogenic because the host is tolerant to the native protein, but not to the same protein containing citrulline. Post-translational modification of antigen has also been implicated in the pathogenesis of T1D [77].

## 5. Animal Models Have Contributed Significantly to Understanding of the Pathogenesis and Treatment of Human Autoimmune Diseases

Understanding of the disease process in autoimmunity has been aided significantly by studies in animal models (Table 2). These models have also been the mainstay of testing of new drug candidates. There is no single animal model that can fully replicate all clinical features of a particular human autoimmune disease. Different rodent models are available for these diseases [57,58,60,61,62,68,78,79,80] (Table 2). Of these, some are experimentally induced, while others are spontaneous in origin. These include, for example, adjuvant-induced arthritis (AA) and collagen-induced arthritis (CIA) for RA; experimental autoimmune encephalomyelitis (EAE) for MS; non-obese diabetic (NOD) mice for T1D, chemical, spontaneous or T cell transfer models of colitis for ulcerative colitis (UC), and Imiquimod-induced psoriasis [57,58,60,61,62,68,78,79,80] (Table 2). Predominantly T cell-driven effector mechanisms are operative in these human autoimmune diseases.

## 6. The Use of Plant-Derived Natural Product Compounds for the Treatment of Autoimmune Diseases

**Figure 2:**
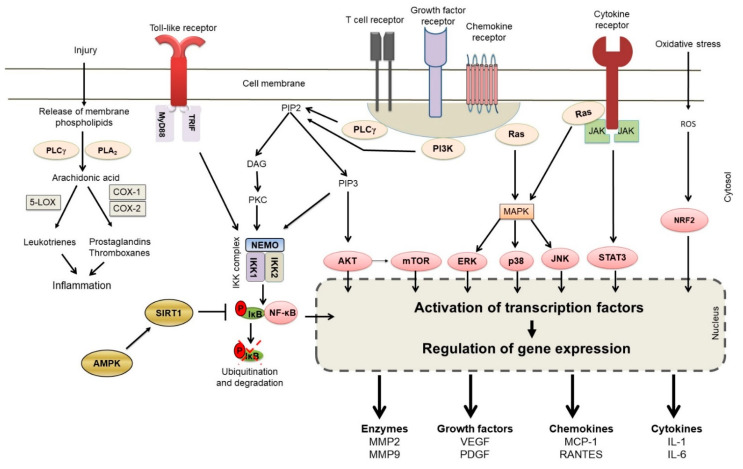
A summary of the main biochemical pathways involved in autoimmune inflammation that are targeted by natural product compounds. Representative mediators of inflammation following modulation of gene expression are shown. (Abbreviations: AKT = an oncogene, also known as protein kinase B or PKB; AMPK = adenosine monophosphate-activated protein kinase; COX; cyclooxygenase, DAG, diacyl glycerol; ERK, extracellular signal-regulated kinase; IKK, IκB kinase; IL = interleukin; IP3, inositol triphosphate; JAK, janus kinase; JNK, c-Jun N-terminal kinase; LO (or LOX), lipoxygenase; MAPK, mitogen-activated protein kinase; MCP-1 = monocyte chemoattractant protein; MMP = matrix metalloproteinase; mTOR, mammalian target of rapamycin; MyD88, myeloid differentiation primary response gene 88; NEMO, NF-κB essential modulator; NF-κB, nuclear factor kappa-light-chain-enhancer of activated B cells; Nrf-2, nuclear factor erythroid 2-related factor 2; PDGF = platelet-derived growth factor; PI3K, phosphatidylinositol-3-kinase; PIP, phosphatidylinositol bisphosphate; PLA_2_, phospholipase A2; PLCγ, phospholipase C gamma; RANTES = Regulated upon Activation, Normal T Cell Expressed and Presumably Secreted; Ras = Ras oncogene; ROS = reactive oxygen species; STAT, signal transducer and activator of transcription; SIRT1 = Sirtuin 1; TRIF, TIR-domain-containing adapter-inducing interferon-β; and VEGF = vascular endothelial growth factor).

The progression of initial autoimmune reactivity to subsequent clinical disease involves inflammation driven by multiple cellular and molecular mediators [53,54,81]. The role of the T cells, macrophages and B cells is briefly discussed above. Various biochemical (e.g., leukotrienes) and immunological (e.g., cytokines) mediators released by these cells, along with others (e.g., complement components), lead to autoimmune inflammation. When uncontrolled, such inflammation can lead to tissue damage and dysfunction. Thus, the control of inflammation is one of the main tenets of treatment of autoimmunity. In this regard, we have described below 5 representative natural product compounds that have extensively been studied for controlling inflammation and re-setting the imbalanced immune system in autoimmune diseases. These include, Resveratrol [19,20,21,22,23,24,25,26], Curcumin [12,27,28,29,30,31], Boswellic acids [15,16,32,33], Epigallocatechin-3-gallate (EGCG) [34,35,36,37,38,39,40], and Triptolide [18,41,42,43,44,45,46]. A summary of these is presented below (Table 3), which also lists the biochemical molecules and pathways targeted by these 5 natural product compounds. The biochemical pathways themselves are outlined in Figure 2. We have focused mostly on the effects of natural product compounds on inflammation in predominantly T cell-driven autoimmune diseases. For space constraints, limited number of studies for each natural product compound are described below.

## 7. Resveratrol

### 7.1. Immune Cells/Response

#### 7.1.1. T Cell Activity and T Cell Subsets

Resveratrol is effective in controlling inflammation by targeting the T cells, by altering T cell differentiation, and by inhibiting the release of pro-inflammatory cytokines and other (biochemical) mediators of inflammation [82]. These outcomes are attributed to its effects on sirtuin-1, adenosine monophosphate-activated protein kinase (AMPK; energy sensor in cells), and nuclear factor-κB (NF-kB) (Table 3) [82,83]. Resveratrol activates sirtuin-1 (a deacetylase) and inhibits acetylation of its target protein p65/relA, which then inhibits NF-kB activity, resulting in reduced production of mediators of inflammation. Similarly, resveratrol targets AMPK, which is an activator of sirtuin-1 via regulating the cellular levels of nicotinamide adenine dinucleotide (NAD+). Another target of sirtuin-1 is peroxisome proliferator-activated receptor gamma coactivator 1-alpha (PGC-1α), which is activated via deacetylation [82]. Interestingly, AMPK is also a target of other natural products, such as curcumin [84], EGCG [85], triptolide [86] and Soy isoflavones [87], demonstrating one of the key anti-inflammatory mechanisms of action of several plant-derived natural compounds.

Th17 is a prominent T cell subset targeted by resveratrol. Resveratrol is an agonist of sirtuin-1 that can cause deacetylation of STAT3 and inhibit its migration into the nucleus to interrupt activation of retinoid orphan receptor gamma t (RORγt) and IL-17A (henceforth, referred to as IL-17) production [21]. This leads to altered balance between Th17 vs. Treg. Another mode of action of resveratrol involves modulation of ligand-induced activation of aryl hydrocarbon receptor (AhR), which in turn suppresses Th17 activity [21]. RORγt also has inhibitory effect on Th1 differentiation and thereby, resveratrol also alters Th1/Th2 balance. Its inhibitory effects on both the pro-inflammatory T cell subsets mentioned above tilt the balance in favor of anti-inflammatory (Th2) and immunoregulatory (Treg) activities. The re-setting of the Th17/Treg cell balance by resveratrol was also evident from a study on immune cells of a patient with a different autoimmune disease, immune thrombocytopenic purpura (ITP) [88]. The observed effects of Resveratrol were attributed primarily to its inhibitory effect on AhR. This was further validated by reversal of the effect of resveratrol by an agonistic ligand for AhR, 2,3,7,8-Tetrachlorodibenzo-p-dioxin (TCDD) [88].

Resveratrol was found to be safe and well-tolerated when administered to healthy male individuals [89]. There was an increase in γδ T cells and Treg in circulation as well as antioxidant activity of plasma when compared with control subjects [89]. However, there was a reduction in pro-inflammatory cytokines TNF-α and MCP-1.

#### 7.1.2. Macrophages and Dendritic Cells (DC)

Resveratrol also controls macrophage activation by lipopolysaccharide (LPS), TLR4 signaling, and other immune activators [82]. These effects are mediated via resveratrol-induced inhibition of NF-kB pathway, COX-2 pathway, TLR expression, as well as NLR family pyrin domain containing 3 (NLRP3)-inflammasome activation [82]. In addition, resveratrol also favors the generation of anti-inflammatory (M2) macrophages.

Aging or senescence is one of the factors affecting self-tolerance and immune reactivity to self-antigens, which can facilitate the induction of autoimmunity [90]. Accumulation of advanced glycation end products (AGEs) produced by nonenzymatic glycosylation of proteins, lipids, and nucleic acids, cause activation of DCs. Resveratrol interferes in the events triggered by the activation of RAGE, the receptor for AGE [90], thus altering the expression of surface maturation markers on DCs and the production of pro-inflammatory cytokines by these cells. These effects involved MAPK and NF-κB pathways [90].

#### 7.1.3. B Cells, Plasma Cells and Autoantibodies

Resveratrol has been shown to have significant effects on the B cells, plasma cells and autoantibody production, all of which play a role in the pathogenesis of antibody/immune complex-mediated diseases, such as lupus [82]. Resveratrol treatment affords protection against lupus, including lupus nephritis, in part via activation of sirtuin-1 and upregulation of inhibitory Fc receptor (FcγRIIB) [82]. The former controls B cell proliferation and autoantibody production, while the latter reduces the activation and number of B cells/ plasma cells in the spleen and bone marrow as a result of apoptosis [82].

### 7.2. Rheumatoid Arthritis (RA)

#### 7.2.1. RA Patients

In pilot clinical trials in RA patients, resveratrol has shown beneficial effects against joint inflammation, pain and other complications associated with RA [91,92,93,94]. In one of these studies on larger numbers of patients, resveratrol was found to be effective against RA, and there it was tested as an adjunct to conventional therapy [94,95]. In some other studies, resveratrol was found to show beneficial effects compared to control groups [95]. These effects were attributed to its anti-inflammatory and antioxidant effects, combined with its effects on the disease-related immune responses [91,95]. These are promising pilot data for larger studies on resveratrol as a potential therapy for RA. Resveratrol can also suppress propagation of joint inflammation by inhibiting the interplay between chondrocytes and macrophages that is triggered by IL-1β, as assessed in a co-culture model system [96]. The treatment of chondrocytes by IL-1β induces IL-6 production via NF-kB activation. This IL-6 activates STAT3 in macrophages, leading to increased IL-6 production [96].

#### 7.2.2. Animal Models of RA

A study in the murine CIA model showed that resveratrol can inhibit clinical arthritis as well as bone loss [97]. Both prophylactic (before disease induction) and therapeutic (after disease onset) regimens of resveratrol administration showed anti-arthritic effects. In parallel, significant effects on the Th17 cell response and anti-collagen antibody levels were observed, with both showing reduced levels/activity compared with controls [97]. In another study in the same model, resveratrol effectively suppressed the development of arthritis in mice [98]. It was associated with inhibition of T cell activation and production of cytokines, which were attributed to resveratrol-induced activation of Sirt1, but reduction of c-Jun acetylation and its migration into the nucleus [98]. Resveratrol treatment of rats with CIA resulted in reduction of paw inflammation, bone loss, cartilage damage and antibody biomarkers (RF and ACPA) [99]. However, there was an increase in the anti-inflammatory cytokine, IL-4.

Resveratrol treatment of arthritic rats having adjuvant arthritis (AA) caused a reduction in paw swelling and biochemical mediators of inflammation, including COX-2 in synovial tissue and prostaglandin E2 (PGE2) level in serum of rats [100]. Prophylactic aspect of resveratrol action against arthritis development was tested in an antigen-induced arthritis (AIA) model [101], and beneficial effects were observed when assessed from paw swelling (clinical effect) along with histology and immuno-histochemistry of paws (reduced synovial hyperplasia and markers of inflammation) [101].

### 7.3. Multiple Sclerosis/EAE

An imbalance between Th17 and Treg cells has also been implicated in the pathogenesis of MS [102]. Accordingly, approaches to enhance Treg generation with or without reduction in Th17 generation are actively being explored. Resveratrol has been shown to facilitate the generation of Treg and thereby to contribute to amelioration of clinical features and underlying immune aberrations in EAE model of MS [102]. Similarly, in vitro generation of Treg has been shown to be enhanced by resveratrol. Multiple mechanisms invoked in this outcome involve resveratrol-induced activation of Sirt1, Estrogen receptor (ER), and AhR [102]. The downstream effects of Sirt1 activation include NF-kB inhibition, which suppresses pro-inflammatory cytokines, and upregulation of Foxp3 via its effect on FoxO3a. Activation of ER and AhR increases Foxp3 expression and IL-10 production, which have immunomodulatory effects in the pathogenesis of EAE and MS [102]. Additional likely pathways proposed for IL-10 production by resveratrol include induction of IL-27 by myeloid cells and activation of the transcription factors cMAF and STAT3 in T cells [102].

The involvement of ER and AhR action was also invoked in apoptosis of activated T cells in the spinal cord in EAE mice [22]. The observed apoptosis correlated with increased expression of Fas and FasL, along with enhanced cleavage of caspases 3, 8 and 9 [22]. In another study on EAE, additional mechanisms were unraveled for the protective effect of resveratrol [103]. There was an increase in the number of T cells secreting both IL-17 and IL-10 (IL-17+/IL-10+) as well as NKT cells that produced IFN-γ in the central nervous system (CNS) tissue [103]. Resveratrol also reduced macrophage-produced cytokines IL-6 and IL-12/23 p40. However, no significant alteration in Th17 cell number was observed.

A mechanism involving micro-RNA (miRNA or miR) for the protective effect of resveratrol against autoimmunity was revealed in a study in the B6 model of EAE [104]. Resveratrol treatment induced miR-124 expression, which inhibits sphingosine kinase 1 gene. Considering that miRNAs are emerging as novel immunomodulators of mediators and pathways of autoimmune and other diseases [105,106], this finding adds a new dimension to the mechanism of action of natural products.

### 7.4. Type 1 Diabetes (T1D)

#### 7.4.1. Diabetes Patients

A pilot study on thirteen T1D patients given resveratrol for two months showed beneficial effects as measured by a panel of markers that document the anti-diabetic and antioxidant effects of this natural product [107]. However, there was not much effect on the levels of insulin and markers of inflammation and functions of liver and kidney. These are encouraging results for further exploration of anti-diabetic effects of resveratrol in a large, controlled study.

#### 7.4.2. Animal Models of T1D

A study in streptozotocin (STZ)-induced diabetes in rats showed that resveratrol afforded protection against T1D development (prophylactic effect) [108]. This effect involved inhibition of apoptosis of pancreatic beta islet cells and suppressed activation of caspase 3 and poly (ADP-ribose) polymerase (PARP). In the STZ model in mice, resveratrol ameliorated the metabolic (biochemical and oxidative stress) aberrations as well as dysregulated immune cell infiltration into pancreatic islets [109]. In another study, resveratrol treatment of diabetic mice reduced the infiltration of macrophages into pancreatic islets as well as the expression of CXCL16/ NF-kB p65 in the islets and spleen compared with untreated mice [109]. One of the complications of T1D involves the reproductive system, specifically reduced spermatogenesis and poor quality of sperms, which affects male fertility. Resveratrol improved both these aspects relating to sperm generation and function by inhibiting sperm DNA damage in rats [110,111].

Resveratrol was also effective in protection against development of T1D in non-obese diabetic (NOD) mouse model [112]. This effect correlated with reduced migration of Th17 cells and macrophages from the peripheral lymph nodes into pancreatic islets. Chemokine (C-C motif) receptor (CCR6) expressed on the surface of both these cell types was one of the targets of resveratrol action [112].

From the foregoing description, it might appear that Th17 and IL-17 are always pathogenic and IL-10 is a typical immunomodulatory cytokine. However, certain microbial components (e.g., Bacillus Calmette-Guérin (BCG) and complete Freund’s adjuvant (CFA)) that can induce IL-17 response may also be protective against inflammation, as elaborated in animal models of T1D [81,113]. In this case, the T cells producing IL-17 and IL-10 (Treg17) show regulatory effects in T1D. Similarly, IL-10 has also been shown to have a pathogenic role under certain disease conditions [114]. Therefore, the outcome of the activity of certain T cell subsets and cytokines can differ in various disease conditions, depending on the timing and level of immune response.

### 7.5. Colitis

Using DSS-induced colitis model, it was shown that resveratrol can induce protection against disease-related clinical symptoms, inflammation and immune pathology [20,115,116]. Resveratrol not only improved inflammation score, but also reduced the infiltration of neutrophils and pro-inflammatory T cells in the mesenteric lymph nodes and lamina propria. Also decreased were markers of inflammation and associated stress, such as p53. In addition, resveratrol enhanced SIRT1 expression, but inhibited COX-2 and NF-kB activities.

A study in TNBS-induced colitis model showed a beneficial effect of resveratrol on inflammation, immune cell infiltration and pathological changes of colitis [117]. There was reduction in Th17 cells but increase in Treg cells, thus altering the Th17/ Treg ratio. Additionally, resveratrol reversed the gut microbial dysbiosis in this model [117], and the microbial components influenced the differentiation of these T cell subsets.

### 7.6. Psoriasis

Studies conducted in the in-vitro (keratinocytes or porcine skin) and in-vivo (imiquimod-induced skin lesions) models of psoriasis showed that resveratrol can inhibit inflammation, reduce pro-inflammatory immune mediators such as cytokines (e.g., IL-1β, IL-6, 17A) and chemokines (e.g., CXCL8), and alter genes relating to retinoic acid stimulation [118,119,120,121]. Furthermore, new formulations of resveratrol, such as nanoemulsion (NE) gel [120] and carbopol gel [119], were found to improve the solubility and skin permeability of resveratrol, and thereby enhance its efficacy while reducing its toxicity. Also tested were natural oligomers of resveratrol, and ε-viniferin was identified as a promising entity for further consideration for a more effective therapy for psoriasis.

### 7.7. Safety and Adverse Effects

The use of resveratrol to control inflammation and disease pathology in various autoimmune diseases, both animal models and patients/volunteers, was generally found to be safe and well-tolerated [89,122,123]. Although uncommon, in some cases, the side effects observed include fatigue, headache, dizziness, gastrointestinal symptoms (e.g., nausea, indigestion, abdominal pain, or vomiting), skin rash or fever. However, there was no evidence of elevated enzymes in blood or other features of hepatotoxicity associated with the use of resveratrol.

## 8. Curcumin

### 8.1. Inflammation/Immune Response

#### 8.1.1. T Cell Activity and T Cell Subsets

Curcumin mediates its anti-inflammatory effects via multiple pathways [27,124,125,126] (Table 3, Figure 2). It inhibits the proliferation as well as IL-2 production by both mouse and human T cells following their activation [127]. Curcumin also suppresses the proliferation and cytokine production by effector memory T (Tem) cells via channel hKv1.3 inhibition [128]. Curcumin also reduces the levels of TLRs on the T cells [129], which has an impact on autoimmune diseases (e.g., EAE) in which the levels of TLRs on the T cells are increased during the disease process. In addition, curcumin can cause apoptotic cell death of activated T cells by inducing enhanced endoplasmic reticulum (ER) stress response and altered mitochondria functional pathways [130]. Furthermore, curcumin-induced immunomodulatory effects can be attributed to the deviation of pro-inflammatory Th1 response to anti-inflammatory Th2 type, inhibition of pro-inflammatory Th17 cells, and upregulation of the generation as well as activity of immunosuppressive Treg cells [131,132]. In regard to Th17 cells, curcumin reduces IL-6 and IL-23 production by DCs and inhibits RORγt expression and IL-17 production in T cells, leading to reduced Th17 differentiation [133]. An altered expression of metabotropic glutamate receptor-4 (mGluR4) on DCs by curcumin might also affect Th17 differentiation [133].

#### 8.1.2. Cytokines

IL-12 and IFN-α/β signaling involves activation of STAT4. Curcumin modulates STAT4 activity in response to these cytokines, resulting in reduced production of IFN-γ, but increased production of IL-10 [134]. The latter has immunomodulatory effects on pro-inflammatory T helper subsets [135]. Curcumin also has significant effects on transforming growth factor-b (TGF-β) signaling. Accordingly, curcumin can have protective effects by controlling inflammation, fibrosis (e.g., in the lungs, colon, and heart), and wound healing in various autoimmune disorders [136].

#### 8.1.3. Dendritic Cells (DCs)

Curcumin reduced the production of pro-inflammatory cytokines IL-12 and IL-23 by DCs and the Th17 response via its action on Nrf2-sensitive heme oxygenase 1 and STAT3 phosphorylation [137]. (Nrf2 is a transcription factor that plays an important role in antioxidant pathways.) Furthermore, unlike the immune-activating function of DCs, tolerogenic DCs manifest anti-inflammatory and immunomodulatory activities, which suppress T cell- and cytokine-driven immune responses. Curcumin facilitates the generation of tolerogenic DCs in response to certain antigens, which otherwise would have induced an immune response [138]. Curcumin mediates this effect via reducing the expression of co-stimulatory and adhesion molecules on DCs and inhibiting their antigen presentation and cellular migration functions [138].

### 8.2. Rheumatoid Arthritis (RA)

#### 8.2.1. RA Patients

Pilot clinical studies in RA patients have revealed beneficial effects of curcumin on clinical symptoms, including joint swelling, pain and discomfort [139,140,141]. These include randomized and controlled studies. A combination of mechanisms relating to curcumin’s anti-inflammatory, antioxidant, and immunomodulatory activities contribute to this anti-arthritic effects. Several of these mechanisms are also discussed below in the context of curcumin’s effects in animal models of RA.

#### 8.2.2. Animal Models

Several studies conducted in different models of RA, including rat adjuvant arthritis (AA) model and mice/rat collagen-induced arthritis (CIA) model, have highlighted the anti-arthritic activity of curcumin. For example, curcumin has been shown to inhibit the development and progression of AA in rats [142,143,144,145], CIA in rats [146], and CIA in mice [147,148,149,150]. Taken together, this arthritis-protective effect is executed at multiple levels of immune response involving macrophages, T cells, B cells, fibroblasts, synoviocytes, osteoblasts, and neutrophils [151,152]. Besides targeting the inhibition of pro-inflammatory cytokines and Th1/Th17 cells, curcumin modulates B cell and antibody responses, including inhibition of IFN-γ-induced BAFF (B cell-activating factor belonging to the TNF family) production [149]. Antibodies such as ACPAs serve both as biomarkers of disease and a mediator of inflammation. Curcumin also enhances apoptosis of macrophages and neutrophils, reduces the proliferation of fibroblasts, and inhibits the formation and maturation of osteoblasts. Among the molecular targets of curcumin are NF-κB, activated protein-1 (AP-1), MAPKs, and COX-2 [151,152]. Curcumin suppresses the effect of IL-1β on chondrocytes, fibroblasts and other cells in the synovial tissue, and thereby controls arthritic inflammation [153]. Furthermore, curcumin can upregulate AMPK, which in turn can activate SIRT1 [84]. Curcumin has also been shown to mediate its effect through the gut-brain axis via its effect on the cholinergic system [148]. Some of the above-mentioned studies revealed the benefit of new formulations (e.g., oil-water nanoemulsion, micellar solubilization, solid nanoparticles, and microencapsulation) to enhance the solubility and bioavailability of curcumin for arthritis therapy [142,143,144,145].

### 8.3. Ankylosing Spondylitis (AS)

Dysregulation of T helper cell/Treg balance has also been observed in AS. Administration of curcumin to AS patients resulted in upregulation of Treg frequency in peripheral blood coupled with cytokine changes [154]. The latter included reduced IL-6, but increased IL-10 and TGF-β production.

### 8.4. Multiple Sclerosis/EAE

A study of EAE in SJL mice showed that the disease-protective effect of curcumin was attributed to inhibition of IL-12-mediated effects on antigen-directed T cell proliferation and differentiation of Th1 cells [155]. These effects were mediated via blocking the JAK-STAT pathway involving JAK2, tyrosine kinase 2 (TYK2), STAT3 and STAT4 [155]. In the EAE model in B6 (=C57BL/6) mice, the disease development was associated with enhanced pro-inflammatory cytokines produced by the APCs (IL-12 and IL-23) and the pathogenic T cells (IFN-γ and IL-17) in both the CNS and the lymphoid tissues. Curcumin treatment suppressed clinical EAE along with inhibition of the above-mentioned cytokines, but upregulation of immunoregulatory cytokine, IL-10 [156]. Also increased were peroxisome proliferator activated receptor γ (PPARγ) and the disease protective Treg cells [156]. In another study, but in rat EAE model, curcumin-induced inhibition of the disease involved reduced proliferation of, and pro-inflammatory cytokine production by, the T cells reactive against myelin-basic protein (MBP) [157]. A prominent effect was observed on Th17 cells and the cytokines (e.g., IL-6, IL-21 and TGF-β) and transcription factors (e.g., STAT3 and RORγt) associated with the differentiation and activity of Th17 cells [157].

### 8.5. T1D

Several studies in murine/rat models of STZ-induced T1D [158,159,160,161] and diabetes in NOD mice [162] have shown the anti-diabetic effects of curcumin. Additional information about the protective effect of curcumin was obtained by using insulinoma cells lines, such as MIN6 [163]. In animal models, curcumin reduced blood glucose levels, improved lipid profile, reduced lipid peroxidation, and improved pancreatic beta cell functions. Taken together, various mechanisms unveiled in these studies demonstrate the anti-inflammatory, antioxidant, and immunomodulatory activities of curcumin [158,159,160,161,162]. For example, curcumin reduced pro-inflammatory cytokines such as TNF-α; decreased ROS; inhibited NF-kB; and decreased Th1 differentiation and activation status of DCs. In cell line-based assays, curcumin reduced apoptosis of beta islet cells by reducing oxidative stress and endoplasmic reticulum (ER) stress [163]. In the animal models, besides its beneficial effects against general metabolic aspects of T1D, curcumin also can provide protection against various complications associated with T1D, such as cardiomyopathy [164], nephropathy [165], and skeletal muscle atrophy [161]. Taken together, these studies have set the stage for clinical testing of curcumin in the treatment of human T1D and its complications.

### 8.6. Colitis

Clinical testing in patients with UC has shown beneficial effects of curcumin [141], which is further supported by mechanistic studies in animal models of colitis described below. In a study based on the DSS-induced colitis model, curcumin treatment showed protection against colitis [166]. Immunologically, this disease-suppressive effect correlated with altered ratio of various subsets of T cells including memory sub-populations. Curcumin not only reduced pro-inflammatory cytokines (IL-7, IL-15, and IL-21), but also re-set the previously dysregulated differentiation and relative balance of naïve, T central memory (Tcm), and T effector memory (Tem) cells [166]. These effects involved inhibition of JAK1/STAT5 signaling. In another study in the same colitis model, curcumin treatment was shown to inhibit the development and propagation of the disease [167]. This outcome involved altered differentiation of T follicular helper (TfH) cells, leading to an imbalance in various subsets of this category of cells [167]. For example, there was reduction in TfH17 and Tem, coupled with upregulation of TfH1, TfH10, TfH21 and Tcm-Tfh. The corresponding cytokines and transcription factors involved in these subsets were also altered accordingly.

### 8.7. Psoriasis

#### 8.7.1. Psoriasis Patients

Psoriasis is an autoimmune disease of the skin characterized by thick, scaly patches, or plaques. Clinical assessment of these plaques is done based on the clinical evaluation of these lesions and presented as the Psoriasis Area and Severity Index (PASI) score. A meta-analysis of 7 clinical studies on the treatment of psoriasis patients with curcumin compared with conventional therapy as well as the combination of the two together showed that curcumin had beneficial effect [168]. There was significant improvement in the PASI score with curcumin alone. However, the effect was not better than conventional treatment alone. Of the 3 modalities, the combination therapy was most effective in controlling the disease symptoms [168]. A few adverse effects observed with curcumin treatment in some patients. These included, nausea, diarrhea, skin rash, and cheilitis [168]. Additional studies on psoriasis patients have also validated the beneficial effects of curcumin [141].

In other studies, examination of the effect of curcumin on PBMC of psoriasis patients showed reduced T cell proliferation and production of cytokines, such as IFNγ, IL-17, GM-CSF and IL-22 [169]. These effects involved an action on the stress-response enzyme heme oxygenase-1. Furthermore, curcumin suppresses the production of IFN-γ by T cells, NK and NKT cells, and IL-17 production by Th17 cells by inhibiting STAT3 phosphorylation in PBMC of psoriasis and psoriatic arthritis patients [170].

#### 8.7.2. Animal Models

Studies in imiquimod-induced murine model of psoriasis have validated the beneficial effects of curcumin in reducing the severity of skin lesions and associated symptoms [171,172,173,174]. Taken together, this protective effect was attributed to reduction of pro-inflammatory cytokines IL-1β and IL-6 [171]; inhibition of IL-6/STAT3 signaling pathway [173]; and suppression of NLRP3 expression, reduction in inflammation caused by cytokines IL-18 and IL-22, and inhibition of IL-22-induced STAT3 activation [174].

### 8.8. Safety and Adverse Effects

Overall, curcumin has been found to be safe in animal models and patients/volunteers. The side effects observed in some individuals include mild and manageable gastrointestinal symptoms (yellow stool, indigestion, or diarrhea), headache, and skin rash have been reported [175,176]. Other effects reported are either infrequent or not fully established [175,176]. For example, gall bladder contraction (squeezing) can occur infrequently, therefore, the use of curcumin in patients with gall stones may require some caution. Similarly, curcumin may increase the risk of bleeding in some cases, and that of uterine contraction in pregnant women. However, no mutagenic or genotoxic effects of curcumin have been observed in animal testing.

## 9. Boswellic Acids

### 9.1. Inflammation/Immune Response

Boswellic acids and its parent herb have been shown to be beneficial in several chronic inflammatory/autoimmune diseases, such as RA, ulcerative colitis, and diabetes [32,177,178]. Boswellic acids, a mixture isolated from gum resin of boswellia serrata, include four major pentacyclic triterpene acids: beta-boswellic acid, 3-acteyl-beta-boswellic acid, 11-keto-beta-boswellic acid (KBA), and acetyl-11-keto-beta-boswellic acid (AKBA) [179]. Among these, KBA and AKBA are widely studied for their biological and medicinal effects [33]. Boswellic acids affect the immune system in multiple ways. For example, boswellic acids inhibit pro-inflammatory cytokines (e.g., TNF-α, IL-1β, IL-2, IL-4, IL-6 and IFN-γ), increase phagocytosis of macrophages, alter antibody production, and inhibit the classical complement pathway [177,178]. The mode of action of boswellic acids differs from that of NSAIDs. Inhibition of 5-lipooxygenase (5-LO or 5-LOX) leading to reduced production of leukotrienes comprises a major activity of boswellic acids, whereas inhibition of prostaglandin synthesis is rather a minor effect (Table 3, Figure 2) [180,181]. Another prominent mode of action of boswellic acids involves inhibition of NF-kB activation [32,182].

#### T Cell Activity/T Cell Subsets

Boswellic acids inhibited the production of pro-inflammatory IL-2 and IFN-γ (both Th1-related cytokines) but upregulated the production of anti-inflammatory/immunomodulatory IL-4 and IL-10 (both Th2-related cytokines) by murine splenic T cells [183]. This Th1 to Th2 switch contributes to the anti-inflammatory and anti-arthritic effects of boswellic acids. In addition, AKBA has been shown to inhibit both Th17 differentiation and the production of cytokine IL-17 by these cells [184]. Two of the mechanisms relating to Th17 differentiation were identified as the targets in this process: inhibition of IL-1β signaling and STAT3 phosphorylation [184].

### 9.2. Rheumatoid Arthritis

#### 9.2.1. RA Patients

Boswellic acids have anti-inflammatory properties and have shown anti-arthritic activity in pilot clinical trials in RA patients [180,185]. Their use was associated with mild side effects. Similarly, boswellic acids have been used to relieve pain in patients with osteoarthritis [182], which is known to have a component of inflammation, although of much lower level than in RA. Furthermore, boswellic acids combined with glucosamine showed synergistic effect, suggesting that similar drug combinations can be explored for clinical applications in arthritis patients and possibly other chronic inflammatory disorders [179,186].

#### 9.2.2. Animal Models of RA

Boswellic acids are known to mediate anti-inflammatory activity when administered systemically. Their similar efficacy after topical application has also been reported in various rodent models of inflammation, such as arachidonic acid and croton oil-induced ear edema and carrageenan-induced paw edema models in mice, and adjuvant-induced arthritis model in rats [179,186].

Boswellia extract containing boswellic acids was tested for anti-inflammatory/anti-arthritic activity in different assay systems, including RAW 264.7 macrophages, human peripheral blood mononuclear cells stimulated with bacterial lipopolysaccharide (LPS), and the rat CIA model [187]. The following mediators were found to be reduced in these assays [187]: TNF-α, IL-6, nitric oxide, and COX-2 secretion; phosphorylated-NF-κB (P65); collagenase, elastase, hyaluronidase, and matrix-degrading enzymes; reactive oxygen species (ROS); circulating anti-collagen antibodies; C-reactive protein (CRP) and prostaglandin E2 (PGE2); and cartilage oligomeric matrix protein (COMP). However, increased levels of hyaluronan were found in the synovial fluid.

Boswellic acids were shown to possess anti-inflammatory activity against AA in rats [188]. Poor solubility and bioavailability of boswellic acids has become a limitation in its use for arthritis and other inflammatory conditions [189]. Attempts are being made to develop novel formulations to enhance their therapeutic index. Micellar formulation has shown improved efficacy over regular preparation of boswellic acids, when tested in the rat AA model [144]. Another study provided insight into increased lysosomal stability following administration of boswellic acids to rats with AA [190,191]. Boswellic acids also revealed their anti-arthritic activity in bovine serum albumin (BSA)-induced arthritis in rabbits, when administered orally or injected into knee joints.

### 9.3. Type 1 Diabetes

Boswellia serrata extract was tested in adult patients with latent autoimmune diabetes [192]. The measurement of the two key biomarkers of this disease, namely antibodies to GAD65 and IA2 antibodies, indicated a beneficial effect of boswellia extract in preventing insulitis and thereby, disease progression at that stage [192]. The results of this pilot study need validation in a larger, controlled study. In another study, boswellic acids were tested in the mouse STZ-model of diabetes [193]. There was reduction of pro-inflammatory cytokines, immune cell infiltration into pancreatic islets, and hyperglycemia. These are promising results for further testing in diabetes patients.

### 9.4. Multiple Sclerosis/EAE

MS and some other neurodegenerative diseases are associated with cognitive deficits [194]. About 70% of MS patients may be affected with such deficit. In a clinical trial on 60 MS patients, treatment with an extract of Boswellia serrata was found to be effective in improving cognition [194]. Specifically, auditory/visual and visual/spatial memory was enhanced in objective tests. In another trial, Boswellia papyrifera showed significant improvement in visuospatial memory [194,195]. This is a promising lead for further investigation of the beneficial effects of AKBA and other boswellic acids for MS therapy.

In a different study, boswellic acids, when tested in a model of EAE in guinea pigs, displayed their effect on reduction of clinical disease [196]. However, the infiltration of immune cells into the CNS was not affected.

### 9.5. Colitis

#### 9.5.1. Colitis Patients

Patients with colitis (UC) were treated with Boswellia serrata gum resin and then various disease-related parameters were assessed [197]. These included, stool examination, rectal biopsy histology and scan microscopy, and blood chemistry and cell counts [197]. All these parameters improved following above treatment. Remission was noted in 82% of treated patients [197]. In another study by the same group, a similar treatment of colitis patients resulted in improvement of clinical symptoms as well as test results in 18 of 20 patients [198]. Of the 20 patients, 14 had remission from the disease symptoms.

#### 9.5.2. Animal Models

A drug delivery system targeted to the colon was developed and tested for the delivery of boswellic acids in a rat model of colitis [199]. One of the objectives of this approach was to limit the action of this natural product to the colon, without any collateral unwanted effects on other gastrointestinal components. This approach resulted in increased bioavailability of boswellic acids in the colon, while sparing other organs such as the stomach, pancreas and liver. Another study revealed the effect of a semi-synthetic AKBA on downregulation of the expression of P-selectin in the colonic tissue, as well as inhibition of P-selectin-mediated cellular interactions with endothelial cells of venules in colonic lesions [200]. Intravital microscopy was used to study these cellular interactions.

### 9.6. Psoriasis

A topical application containing boswellic acids was tested in patients with psoriasis [201]. Readouts consisted of changes in scales and extent of erythema at the site of lesions. Both parameters showed improvement by topically applied formulation. In a different study, efficacy of boswellic acids was assessed using a structural bioinformatics approach in the context of psoriasis treatment [202]. First, key potential targets of psoriasis were selected. Then, structure-based screening was performed to identify the compounds that represented potential targets for psoriasis therapy. These included, for example, TNF-α, IL-17, eNOS, iNOS, Janus Kinases (JAKs), and MAPK [202]. The in-silico results correlated well with experimental observations.

### 9.7. Safety and Adverse Effects

The use of AKBA or other common boswellic acids has been found to be generally safe [180,185]. Adverse effects were rarely observed in clinical trials or animal model studies [203,204]. However, in some cases, gastrointestinal symptoms (e.g., nausea, diarrhea or abdominal pain) have been reported, but these were mild and manageable.

## 10. EGCG

### 10.1. Rheumatoid Arthritis

Green tea polyphenols (GTP), when administered orally to mice, resulted in reduced incidence as well as severity of CIA [35]. This was associated with reduced expression levels of COX-2, TNF-α, IFN-γ, and anti-collagen antibodies, but increased activity of neural endopeptidase in the joints of mice. Subsequent studies in the CIA model further validated the disease-protective effects of EGCG. In one such study, EGCG treatment of obese CIA mice showed its effectiveness in suppressing arthritis progression [205]. It correlated with reduced Th17 combined with increased Treg, and inhibition of STAT3. In another study, the treatment of CIA mice with EGCG was shown to ameliorate progression of clinical arthritis [206]. This outcome correlated with altered Th17/Treg balance in favor of the latter, along with suppression of osteoclastogenesis [206]. These effects involved EGCG-induced upregulation of the activity of phosphorylated-ERK, Nrf2, and HO-1, coupled with downregulation of STAT3 activation. Another aspect of anti-inflammatory activity of EGCG involves activation of AMPK, which can then activate SIRT1 [85]. This action of EGCG is similar to that of resveratrol and curcumin described above.

The testing of EGCG in RA synovial fibroblasts in culture as well as rat adjuvant-induced arthritis (AA) model have unveiled novel aspects of action of this natural product. Exposure to EGCG of IL-1β-treated RA synovial fibroblasts resulted in inhibition of several chemokines, including RANTES, epithelial-derived neutrophil-activating peptide 78 (ENA-78), GROα, MCP-1 [34]. Also reduced was MMP2 activity. These outcomes were attributed to inhibitory effects of EGCG on PKCδ phosphorylation and NF-kB activation. In another study, EGCG treatment of rats with AA caused suppression of clinical disease coupled with reduction of IL-6 in serum and joints [207]. Using RA synovial fibroblasts, EGCG was shown to inhibit IL-1β-induced IL-6 production and increased soluble gp130 (sgp130) production. The latter effect was attributed to trans-signaling via induction of alternative splicing of gp130 mRNA [207]. Increased sgp130 synthesis was proposed to inhibit IL-6/soluble IL-6R-induced MMP2 activity in RA synovial fibroblasts and joint tissue of rats. In a different, prophylactic study in the rat AA model, green tea extract containing EGCG was shown to afford protection against arthritis development [208]. This treatment reduced pro-inflammatory cytokine IL-17 but increased immunomodulatory cytokine IL-10 production by the draining lymph node cells [208]. In addition, the level of antibodies to mycobacterial heat-shock protein 65 (Bhsp65), a disease (arthritis)-related antigen, was reduced.

The testing of EGCG in interleukin (IL)-1 receptor antagonist knockout (IL-1RaKO) arthritis model showed its inhibitory effect against clinical arthritis, bone damage, and osteoclast activity [209]. In addition, EGCG decreased the levels/activity of pro-inflammatory cytokines, oxidative stress proteins, p-STAT3, mTOR and HIF-1α [209]. Furthermore, the effects on mTOR and HIF-1α correlated with altered Th17/Treg balance.

A limited number of pilot clinical studies have indicated protection by EGCG against cardiovascular and rheumatic complications in RA [210,211], but larger clinical trials are needed to validate these claims.

### 10.2. Sjogren’s Syndrome (SS)

SS is characterized by immune cell infiltrates into the salivary and lacrimal glands, leading to their destruction and dysfunction. In a study in the NOD mouse model of human SS, EGCG treatment reduced the levels of autoantibodies as well as immune cell infiltrates into the submandibular glands [212]. EGCG also ameliorated TNF-α-induced cytotoxicity of acinar cells, which involved p38 MAPK phosphorylation-based activation.

### 10.3. Multiple Sclerosis/EAE

The protective effect of EGCG against EAE in B6 model was shown to involve the production as well as cell signaling events related to cytokines IL-2, IL-15, and IL-7, which share the physiological function of regulating the survival, expansion, and differentiation of T cells [213]. EGCG also inhibited the expression of receptors for these 3 cytokines. The effect of EGCG on other cytokines and T cell subsets in EAE was revealed in additional studies. For example, in one study, EGCG was effective in inhibiting the progression of EAE by reducing the production of IFN-γ and IL-17 by CD4+ T cells and inhibiting the differentiation of these two pathogenic T cell subsets [214]. It involved modulation of the T cell subset-specific transcription factors, T-bet and RORγt, respectively, along with that of the STAT pathway [214]. EGCG also suppressed the activity of antigen-presenting cells by reducing their costimulatory function mediated via CD80 and CD86. In another study, EGCG treatment of EAE mice afforded protection against clinical disease and immune pathology [215]. These beneficial effects involved reduction in CD4+ T cell activation and proliferation, along with suppression of both pro-inflammatory cytokine production and the frequency of Th1 and Th17 cells [215]. However, there was an increase in Treg cells. The impact of EGCG on macrophages was unveiled in another study where EGCG treatment was shown to reduce macrophage infiltration in the CNS and the spleen, which was associated with reduced disease severity, [216]. Furthermore, EGCG was able to alter M1/M2 ratio in favor of the latter, when tested in vitro, which was attributed to NF-kB inhibition and glycolysis.

### 10.4. T1D

The anti-diabetic effects of EGCG were revealed through in vitro and in vivo testing. In vitro, EGCG treatment prevented cytokine-driven destruction of RINm5F cells (an insulinoma cell line) and restored insulin secretion [217,218]. Reduction in the activity of nitric oxide synthase (NOS), apparently via NF-kB inhibition; suppression of the cytokine-induced generation of reactive oxygen species (ROS); and modulation of the mitochondrial pathway were involved in this protective effect of EGCG. In vivo, EGCG treatment of STZ-induced T1D [217] reduced the blood glucose level, downregulated iNOS, and reduced the islet cell mass [218]. In the NOD mouse model, EGCG treatment resulted in delayed onset of diabetes, but without any effect on insulitis per se [219]. The level of insulin and glycosylated hemoglobin in plasma was reduced, whereas the level of immunomodulatory cytokine IL-10 was increased [219].

### 10.5. Colitis

Several studies conducted using the TNBS-colitis and DSS-colitis model showed anti-inflammatory, immunomodulatory, and antioxidant effects of EGCG. These beneficial effects were evident in the form of inhibition of clinical and histological manifestations of colitis along with suppression of biochemical and/or immunological mediators of inflammation. Taken together, different studies revealed the following mechanisms of action of EGCG: (a) Reduction in myeloperoxidase (MPO) activity, and inhibition of NF-kB and activator protein-1 (AP-1) [220]; (b) potent anti-oxidant activity as shown by altered levels of tissue concentration of specific mediators [221], such as reduced malondialdehyde (an indicator of lipid peroxidation) and myeloperoxidase (an indicator of neutrophils accumulation), but increased antioxidant enzymes (superoxide dismutase and glutathione peroxidase); (c) reduced pro-inflammatory cytokines TNF-α and IL-6, decreased serum amyloid A (an inflammatory marker) and improved anti-oxidant activity [222]; (d) reduced levels of IL-6 and IL-17, but increased levels of IL-10 and TGF-β1 in the plasma; altered levels of Th17/Treg in favor of the latter in the spleen; and a decrease in HIF-1α and STAT3 protein expression in the colon tissue [223]; (e) decreased levels of IL-1β, IL-6, TNFα, and lipid peroxides in the colonic tissue, coupled with decreased gastrointestinal permeability [224]; (f) reduced production of pro-inflammatory mediators and restored balance of Th1/Th2 [225], which are mediated via the TLR4/MyD88/NF-κB pathway [225]; (g) reduced pro-inflammatory cytokines TNFα, IL-6 and MCP-1 in the colonic tissue, decreased intestinal permeability, and suppressed infiltration of T cells and macrophages into the colonic mucosa [226]; (h) increased levels of bacteria Akkermansia, which produces short-chain fatty acids (SCFAs) [227]; this was further validated by fecal microbiota transplantation (FMT) from EGCG-treated mice to recipient mice that induced protection against colitis in the recipient mice [227]; and increased anti-oxidant capacity and reduced Th1 response in 18-month old mice [228].

### 10.6. Psoriasis

Studies on the testing of EGCG by topical application in imiquimod-induced model of psoriasis-like skin lesions have revealed its beneficial effects [229,230]. The outcomes were assessed by multiple parameters: examination of dermal lesions for skin thickness, erythema and scales; immune cell infiltration in the skin and markers of differentiation (e.g., keratin-10, filaggrin); the ratio of different immune cells in the spleen; the levels of cytokines, chemokines, and indicators of anti-oxidant activity (e.g., malondialdehyde, superoxide dismutase and catalase) in the blood. One of these studies used regular EGCG [230], while the other was based on a nanoparticle formulation of EGCG [229]. The latter was found to improve bioavailability of EGCG significantly when tested in vitro on keratinocytes or in vivo in IIP model mentioned above. These results support large-scale testing of EGCG in patients with psoriasis and exploring nanoformulations further to improve the pharmacokinetics aspects of EGCG.

### 10.7. Safety and Adverse Effects

EGCG is a component of green tea, which is one of the most widely consumed beverages in the world. The use of EGCG in studies in animal models and patients/volunteers has revealed that overall, it has a good safety profile. However, in some cases with underlying diseases such as diabetes and colitis, EGCG may display unexpected adverse reactions [231,232]. For example, EGCG was shown to induce nephrotoxicity or cardiotoxicity in animal models of diabetes and renal toxicity in an animal model of colitis [231,232]. In addition, hepatotoxicity has also been linked with EGCG use in some cases.

## 11. Triptolide

Triptolide mediates its effects on multiple cell types via inhibiting the XPB subunit of the transcription factor TFIIH core complex, which inhibits RNA polymerase II-mediated transcription and repair [233,234]. Specific targets related to inflammation that are identified include, NF-kB, IL-6/STAT3/SOCS3 signaling pathway, MAPK, and AMPK [86,234,235] (Table 3, Figure 2).

### 11.1. Immune Response

Triptolide has immune-inhibitory effects, which are mediated via its effects on the T cells, DCs, and B cells [41,236,237]. In one study, the efficacy of triptolide in suppressing the proliferation and cytokine production (e.g., IL-2 and IFN-γ) of human T cells was compared with that of a well-known immunosuppressant drug, FK506 [236]. Triptolide was found to be superior to FK506 for inhibiting T cell proliferation and IFN-γ production, but comparable to FK506 for IL-2 production. In another study using human monocyte derived DCs, it was shown that triptolide inhibits the maturation and migration of DCs [41]. This was inferred from the in vitro testing of the phenotype of DCs (including cytokine production and chemotaxis in response to defined chemokines), as well as in vivo testing in mice of the migration of Langerhans cells from the skin to the regional lymph nodes [41].

### 11.2. Rheumatoid Arthritis

#### 11.2.1. RA Patients/RA Synovial Fibroblasts

Synovial fibroblasts play a vital role in arthritis pathogenesis, and novel approaches are being tried to suppress their activity and migration in an effort to control the disease process. In a study on synovial fibroblasts from RA patients (RA-FLS), triptolide was shown to inhibit the viability and migration of these cells, and to reduce the production of pro-inflammatory cytokines, but to increase anti-inflammatory cytokine levels [238]. Also inhibited was the activation of JAK-STAT pathway. In another study, the profiles of gene expression and cellular signaling pathways were examined in the synovial tissues of patients with RA and healthy controls [239]. Among several gene groups, CD2 (a T cell surface antigen) was identified and shown to be the target of a triptolide derivative.

Angiogenesis is an integral component of the pathogenesis of RA. TwHf (the parent herb for triptolide) has been shown to inhibit arthritis severity and progression of disease in RA patients and animal models [240]. It inhibits angiogenesis, highlighting one of the several mechanisms of its anti-arthritic activity.

#### 11.2.2. Clinical Trials on TwHF in RA Patients

Several well-controlled clinical trials, including randomized, double-blind, and placebo-controlled trials using the plant extract of TwHF and RA patients have been conducted by several investigators [43,241,242,243,244,245]. TwHF was administered to patients either alone or in combination with a conventional disease-modifying anti-rheumatic drug (DMARD), such as methotrexate. Collectively, these studies concluded that TwHF was effective in reducing the severity of symptoms of RA [43,241,242,243,244,245]. The observed effects were similar to, or in some cases, superior to that of mainstream drugs. However, the use of TwHF was associated with a few adverse reactions pertaining to gastrointestinal tract and hematopoiesis. Apparently because of the adverse effects, the widespread global use of TwHF has not yet materialized. It is hoped that modifications in the drug delivery methods or finding a suitable combination of TwHF with a mainstream drug might help to limit the dose of TwHF required for efficacy, and thereby, permitting its use as a useful adjunct to conventional drugs. Additional trials using triptolide in RA patients would help in fully assessing its relative merits or limitations in comparison with TwHF extract.

#### 11.2.3. Animal Models of RA

Using the rat CIA model, it was shown that triptolide can inhibit clinical arthritis and reduce the level of pro-inflammatory cytokine IFN-γ in the peripheral lymphoid tissue [246]. However, the level of immunoregulatory cytokine TGF-β in the periphery was enhanced. The above alterations correlated with reduced numbers of CD4 and CD8 T cells in Peyer’s patches, the site of immune tolerance, and reduced CD4 numbers in the periphery [246].

Triptolide affects a variety of innate immune cells, which influence various downstream and inter-connected immune pathways in arthritis and other autoimmune diseases [247]. A study based on gene expression and bioinformatics analysis pointed to triggering receptors expressed on myeloid cells (TREM)-1 signaling pathway as a major target [248]. Also reduced were DNAX-associated protein (DAP)12, JAK2, and STAT3. The levels of pro-inflammatory cytokines TNF-α, IL-1β and IL-6 were also reduced [248]. Above changes induced by triptolide were observed in both in vitro and in vivo (in ankle tissue of arthritic rats with CIA) model systems.

In a study in the rat AA model, it was shown that triptolide treatment resulted in inhibition of the disease [249]. The effects of triptolide were also tested on autophagy and cytokine production in the tissues (e.g., synovial tissue, thymus and spleen) [249]. Autophagy plays an important role in a variety of physiological and pathological processes, including RA. The key genes involved in promoting autophagy were suppressed by triptolide, whereas certain pro-inflammatory cytokines tested were found to be reduced.

### 11.3. Multiple Sclerosis/EAE

The protective effect of triptolide against EAE was revealed when tested in B6 model of MS [250]. The onset of the disease as well as its progression was delayed by triptolide, and changes in clinical signs were validated histologically in the CNS tissue [250]. The levels of pro-inflammatory cytokines were reduced in the spinal cord and peripheral lymphoid tissue. The latter also showed enhanced expression of Foxp3, a marker of Treg. Biochemically, the action of triptolide involved inhibition of NF-kB activity and reduced proportion of phosphorylated IkBα [250].

The beneficial effect of triptolide against EAE was also shown in PLP-induced EAE model in SJL/J mice [42]. This protective effect was observed in both prophylactic and therapeutic regimen, and it was associated with marked increase in heat shock protein 70 (Hsp70) in the central nervous system tissue of triptolide-treated mice compared with control mice [42]. In parallel, triptolide inhibited NF-kB activity. It was proposed that Hsp70 directly interacts with NF-kB/IkBα complex and causes its stabilization, which in turn contributes to suppression of inflammation observed in triptolide-treated animals.

### 11.4. T1D

Diabetic cardiomyopathy is a complication of diabetes. The pathogenesis of this myocardial fibrosis involves autoimmune inflammation that is driven in part by TLR4 and its downstream mediator, NF-kB [251]. In a study in rats with diabetes, triptolide treatment was shown to induce protection against myocardial fibrosis and the associated impaired left ventricular function by suppressing both the TLR-4 and NF-kB pathways. This treatment caused reduction in inflammation along with improved cardiac pathology and function [251]. In a study in NOD mice, a model of T1D, triptolide administration reduced the incidence of T1D, which was associated with decreased pro-inflammatory cytokines expressed in pancreatic islets [252]. Furthermore, the proportion of CD4, CD8, and T cells producing IFN-γ in islets were reduced, and triptolide also inhibited NF-kB activity [252].

### 11.5. Colitis

Several studies in two different models of colitis have unveiled the beneficial effects of triptolide. The two models used were DSS-induced colitis [253,254,255,256,257] and spontaneous colitis in IL-10-deficient mice [258,259,260]. Investigations based on the DSS-colitis model showed that triptolide mediates its disease-protective effects via diverse mechanisms [253,254,255,256,257]. These mechanisms include, inhibition of IL-1β in the colonic tissue [257]; suppression of IL-6 expression in the mucosa [256]; recovery from disordered composition of gut microbiota and establishing an optimal balance of Bacteroides vs. Firmicutes [255]; reduced ROS production, M1 polarization, and immune cell infiltration into the mucosa, which involved effect of triptolide on Nrf2/HO-1 signaling as well as PDE4B/Akt/NF-kB pathway [254]; and inhibition of Th1 and Th17 differentiation, which involved targeting of JAK-STAT pathway by a triptolide derivative, ZT01 [253]. Another set of studies on triptolide based on IL-10-deficiency-colitis model indicated the following mechanisms in its beneficial effects [258,259,260]: the targeting IL-17 and inhibition of IL-6/STAT3 pathway [258]; reduction of pro-inflammatory cytokines (e.g., IL-12, IL-23, TNFα, IFN-γ) in colonic tissue, which involved NF-kB inhibition [259]; and inhibition of immune cell infiltration into the mucosa, along with reduced TNFα, IFN-γ, and TNF receptor 2, and NF-kB activity [260].

### 11.6. Psoriasis

#### 11.6.1. Patients

Both triptolide and its parent herbal extract of TwHF have been found to be effective in the treatment of patients with psoriasis [261]. The patients were diagnosed with different clinical phenotypes, including plaque psoriasis, pustular or erythrodermic psoriasis or psoriatic arthritis.

A group of patients with psoriasis vulgaris were treated with triptolide in a tablet form [262]. The outcome measure used was Psoriasis area severity index (PASI). This treatment had marked effect in 41 out of 103 (39.7%) patients, while improvement was observed in another 37 (35.8%) patients [262]. The remaining 25 (24.5%) failed to respond. In regard to the adverse effect, reduced white blood cell counts were recorded in a few patients [262].

#### 11.6.2. In Vitro Model

A mechanistic study on triptolide action was performed on IL-22-treated HaCaT cells, an in vitro model for keratinocytes in pathological lesions of psoriasis [263]. Triptolide inhibited IL22-induced proliferation of HaCaT cells by causing cell cycle arrest, but it promoted the differentiation of these cells.

### 11.7. Safety and Adverse Effects

Despite the beneficial effects of triptolide in arthritis and other autoimmune diseases, its clinical application has been impeded by concerns regarding its side effects [43,86,241,242,243,244,245,264]. The adverse effects were observed in animal models and patients/volunteers. These pertained to gastrointestinal tract, liver, kidney, heart and reproductive organs [43,86,241,242,243,244,245,264,265]. Furthermore, adverse effect on the reproductive system in both males and females is one of the primary concerns. In clinical trials, in some cases, reduced sperm count and functionality in men, and effects on menstruation in women were observed.

## 12. Concluding Remarks

Natural products derived from diverse dietary and medicinal plants are a promising resource for potential therapeutic compounds for the management of chronic inflammatory and autoimmune diseases. The foregoing discussion highlighted mechanisms of action and applications of five such representative compounds in animal models of autoimmunity. In many parts of the world, several natural product extracts are already being used by practitioners of traditional medicine. Similarly, some of the isolated/purified compounds are consumed as over-the-counter food supplements by healthy individuals worldwide for improving immunity and health maintenance. It is hoped that following well-controlled clinical trials in patients with specific autoimmune diseases of interest, one or more of these compounds with desirable traits would be usable globally as therapeutic agents in near future. Probably, these compounds might initially be used as adjuncts to conventional medications before they are considered as alternative choices for certain drugs and diseases. These decisions might differ for different populations based on their prior knowledge and/or experience in the use of natural products for treatment purposes. Nevertheless, natural product compounds offer a vast opportunity for the development of promising new drugs for the treatment of autoimmune diseases.

## Figures and Tables

**Table 1:** Salient features of the major T cell-mediated human autoimmune diseases.

Name	Main Organs Affected *	Genetic Susceptibility	Self-Antigens Targeted	Drugs Used	References
		(e.g., HLA allele)	(representative)	(examples)	
Rheumatoid arthritis (RA)	Synovial joints	DRB1 * 01/* 04/ * 10	CII, Hsp65	NSAIDs, DMARDs	[55]
Multiple sclerosis (MS)	Central nervous system	DRB1 * 1501	MBP, PLP	IFN-β, Glatiramer, Fingolimod	[63,64]
Type 1 Diabetes	Pancreatic β-islets	DRB1 * 0301/ * 0401	Insulin, GAD65	Insulin	[65]
(T1D)					
Ulcerative colitis (UC)	Colon	HLA ADCY7	Gut microbial antigens	Anti-TNF	[66,67]
Psoriasis	Skin	HLA-Cw6 (PSORS1)	Cathelicidin LL-37 ADAMTSL5	Anti-TNF, MTX	[68]

* other organs may also be affected to varying extent in different diseases. HLA = human leukocyte antigen, CII- type II collagen, Hsp65 = heat-shock protein 65, MBP = myelin-basic protein, PLP = proteolipid protein, GAD = glutamic acid decarboxylase, NSAIDs= non-steroidal anti-inflammatory drugs, DMARDs = disease-modifying anti-rheumatic drugs, IFN-beta= interferon-β; ADCY7 = adenylate cyclase 7 gene; LL-37 = cathelicidin peptide; ADAMTSL5 = melanocyte antigen, a disintegrin and metalloprotease domain containing thrombospondin type 1 motif-like 5; MTX = methotrexate; TNFα= tumor-necrosis factor alpha.

**Table 2:** Animal models of the major T cell-mediated human autoimmune diseases.

Disease Model	Strain of Rat/Mouse	Mode of Induction	References
Adjuvant-induced	Lewis rats	Mtb (H37Ra)	[78]
arthritis (AA)			
Collagen-induced	DBA/1 mice	Type II collagen (CII)	[35,79]
arthritis (CIA)	HLA.DR4 mice	CII	
	Lewis rats	CII	
Experimental autoimmune	Lewis rats	MBP, MBP peptide	[22,61,62]
encephalomyelitis (EAE)	SJL mice	PLP, PLP peptide	
	(SWR x SJL) F1 mice	PLP, PLP peptide	
	C57BL/6 mice	MOG, MOG peptide	
Type 1 diabetes (T1D)	C57BL/6 mice	STZ	[27,57]
	NOD mice	Spontaneous	
Colitis	C57BL/6, BALB/c mice	DSS	[20,27]
(Ulcerative colitis; UC)	C57BL/6, BALB/c mice	TNBS	
	IL-10-deficient mice	Spontaneous	
	RAG-deficient mice	CD4^+^ CD45RB^hi^ transfer	
Psoriasis	BALB/c	Imiquimod	[80]

Mtb—heat-killed *Mycobacterium tuberculosis* H37Ra, CII- type II collagen, MBP—Myelin basic protein, PLP—Proteolipid protein, MOG—Myelin oligodendrocyte protein, DSS—Dextran sodium sulphate, STZ—Streptozotocin, TNBS—2,4,6-trinitrobenzene sulfonic acid, RAG—recombinase activating gene.

**Table 3:** Select natural product compounds from plants: their origin, basic chemical characteristics, and targeted molecules and pathways for the treatment of autoimmune diseases.

Product	Plant Sources	Chemistry ^#^	Targets/Pathways ^$,^*	References
Resveratrol	*Vitis vinifera*,	Stilbene	**AMPK**, COX-2, MAPK	[19,20,21,22,23,24,25,26]
	*Polygonum*	C14H12O3	NF-kB, **Nrf2**,	
	*cuspidatum*	MW 228.24	PI3K/Akt/mTOR,	
			SIRT-1, STAT3, **AhR**	
			Wnt/β-catenin, PPARγ,	
Curcumin	*Curcuma longa*	Diarylheptanoid	NF-kB, COX-2, **AMPK**	[12,27,28,29,30,31]
		(beta-diketone)	STAT3, Nrf2, HO-1	
		C21H20O6	MAPK, **JNK**	
		MW 368.4	ROS metabolic pathway	
Boswellic	*Boswellia*	Pentacyclic	5-LO, Akt, COX-2	[15,16,32,33]
Acids	*serrata*	terpenoids	MAPK, NF-kB, **Nrf2**	
(e.g., AKBA)		C32H48O5	STAT3, ERK1/2	
		MW 512.7	MMP9, VEGF	
EGCG	*Camellia sinensis*	Catechin	COX-2, NF-kB, **AMPK**	[34,35,36,37,38,39,40-]
		C22H18O11	AP-1, MMP 2 & 9,	
		MW 458.4	HIF-1a, iNOS	
			PI3K/Akt/mTOR,	
			STAT3, MAPK	
Triptolide	*Tripterygium*	Diterpenoid	XPB/TFIIH	[18,41,42,43,44,45,46]
	*wilfordii* Hook F.	triepoxide	NF-kB, MAPK	
	(TwHF)	C20H24O6	IL-6/STAT3, **SOCS3**	
		MW 360.4		

^#^ MW = molecular weight. ^$^ Targets in bold face font = upregulated; Targets in regular font = downregulated (this direction of change is representative and based on common anti-inflammatory activity of these natural product compounds, but subtle differences can be observed under different contexts of the dose, timing and underlying pathology involved). * Abbreviations: AhR = aryl hydrocarbon receptor; AKBA = acetyl-11-keto-beta-boswellic acid; AMPK = adenosine monophosphate-activated protein kinase; Akt = an oncogene, also known as protein kinase B or PKB; AP-1 = activator protein-1; COX = cyclooxygenase; EGCG = epigallocatechin-3-gallate; ERK = extracellular signal-regulated kinase; HIF= hypoxia-inducible factor; HO-1 = heme oxygenase-1; IKK = IκB kinase; iNOS = inducible nitric oxide synthase; JAK, janus kinase; JNK = c-Jun N-terminal kinase; LO (or LOX), = lipoxygenase; MAPK= mitogen-activated protein kinase; MMP = matrix metalloproteinase; mTOR = mammalian target of rapamycin; NF-κB = nuclear factor kappa-light-chain-enhancer of activated B cells; Nrf-2 = nuclear factor erythroid 2-related factor 2; PI3K = phosphatidylinositol-3-kinase; PPARγ = peroxisome proliferator activated receptor γ; ROS = reactive oxygen species; STAT = signal transducer and activator of transcription; SIRT1 = Sirtuin 1; SOCS = suppressor of cytokine signaling; TFIIH = transcription factor IIH; VEGF = vascular endothelial growth factor; XPB = XPB subunit of TFIIH.

## Data Availability

Not applicable.
